# Defective Bismuth Oxide as Effective Adsorbent for Arsenic Removal from Water and Wastewater

**DOI:** 10.3390/toxics9070158

**Published:** 2021-07-02

**Authors:** Ramona Balint, Mattia Bartoli, Pravin Jagdale, Alberto Tagliaferro, Abdul Samad Memon, Massimo Rovere, Maria Martin

**Affiliations:** 1 Department of Agricultural, Forest and Food Sciences, University of Turin, Largo Paolo Braccini 2, 10095 Grugliasco, Italy; maria.martin@unito.it; 2 Geological Institute of Romania, Strada Caransebes nr. 1, Sector 1, 012271 Bucharest, Romania; 3 Center for Sustainable Future Technologies, Italian Institute of Technology, Via Livorno 60, 10144 Turin, Italy; Pravin.Jagdale@iit.it; 4 National Consortium for Materials Science and Technology (INSTM), Via G. Giusti 9, Florence 50121, Italy; alberto.tagliaferro@polito.it (A.T.); massimo.rovere@polito.it (M.R.); 5 Department of Applied Science and Technology, Polytechnic of Turin, Corso Duca degli Abruzzi 24, 10129 Turin, Italy; abdul_samad_0608@yahoo.com

**Keywords:** water treatment, arsenic, bismuth oxide, adsorption

## Abstract

In this work, we report solid-state synthetized defective Bi_2_O_3_ containing Bi(V) sites as effective and recyclable arsenic adsorbent materials. Bi_2_O_3_ was extensively characterized, and structure-related adsorption processes are reported. Both As(V) and As(III) species-adsorption processes were investigated in a wide range of concentrations, pH values, and times. The effect of several competing ions was also tested together with the adsorbent recyclability.

## 1. Introduction

Water shortage is one of the most concerning threats envisaged for the future, given the growing population and the changing climate [1]. Enhancing the availability of clean water by efficiently removing natural or anthropic contaminants is therefore of paramount importance. Wastewater management is another essential link in reducing the degradation of the environment and improving the sustainability of productive systems [2].

Some of the most dangerous and difficult-to-avoid pollutants affecting water quality are the potentially toxic elements (PTE), ubiquitous in both developed [3,4] and developing countries [5,6]. Among PTE, arsenic is one of the most diffused and dangerous contaminants [7], with varying toxicity levels according to its chemical speciation. In general, the inorganic species, arsenite and arsenate, are considered more toxic than the organic ones [8]. Furthermore, arsenite is more toxic and more mobile in the environment than arsenate [9], which poses further challenges for its removal from water compared to arsenate, requiring, e.g., preliminary oxidation steps in water treatment plants. High levels of As (above 10 µg L^−1^) in groundwater used for drinking and irrigation are not infrequent in southeast Asia, where endemic As poisoning has caused long-term health effects and a public health emergency [5,6,7].

Nowadays, a number of different technologies are available for arsenic removal from groundwater and wastewater, including reverse osmosis [10], ion exchange [11], precipitation [12] and adsorption [13]. Among these, the techniques based on As adsorption are preferred because of their efficiency, economic feasibility and wide adsorbent availability [14] (e.g., oxides and oxy(hydr)oxides of Fe, Al, Ti, and Mn [15,16]; double layered hydroxides [17]; and carbon- [18,19,20] and silica-modified [21,22] surfaces). The key points to be considered for the choice of the adsorbent include its adsorption efficiency and the economic and environmental sustainability of its production and use. To this last point, the potential for adsorbent regeneration deserves great attention since the plants for contaminant removal may generate great amounts of solid waste as contaminant-saturated media, which is hazardous for humans and the environment and requires high costs for disposal [23]. For these reasons, continuing the research aimed at developing new high-performance adsorbents for water treatment is essential. Research has been focusing lately on innovative substrates for the removal of contaminants from water, with bismuth-based materials standing out due to being safe, non-toxic and non-carcinogenic [24]. Bi-based compounds have a number of biomedical applications [25] and display great potential for photocatalytic degradation of organic contaminants [26] and for the removal of different anionic species often found in groundwater and wastewater (e.g., PO_4_^3−^, NO_3_^−^, SO_4_^2−^, Cl^−^, and F^−^) [27]. These materials have been proposed as possible candidates for As adsorption only recently [28,29], with promising results depending on material composition and synthesis method. Wang et al. [29] tested As(III) and As(V) adsorption on Bi-based substrates with promising results, and Zhu et al. [30] described an innovative approach based on bismuth doped biochar for effective arsenic removal showing the promising properties of bismuth based material for the removal of aqueous inorganic pollutants. In this research, the authors suggested an adsorption mechanism based on both surface interactions and electrochemical processes.

In this work, we report the use of surface defective bismuth oxide as an effective adsorbent to remove arsenite (As(III)) and arsenate (As(V)) species from water solutions while considering the influence of pH, contact time, and competitive ions and testing the potential for adsorbent regeneration, together with an extensive characterization of the bismuth oxide material.

## 2. Materials and Methods

### 2.1. Synthesis and Characterization of Bismuth Oxide (Bi_2_O_3_)

Bismuth oxide (Bi_2_O_3_) was synthesized through solid-state reaction by direct heating of bismuth nitrate (Bi(NO_3_)_3_·5H_2_O, 99% purity, Sigma Aldrich, St. Louis, MO, USA) at 150 °C for 30 min for dehydration, followed by 2 h at 250 °C. The material was subsequently annealed at 550 °C for 2 h and finally cooled gradually until reaching room temperature.

X-ray diffraction (XRD) patterns of the synthesized Bi_2_O_3_ were measured with a Pan’Analytical X’Pert Pro diffractometer (Pan’Analytical, Almelo, The Netherlands) equipped with a Cu Kα source. The spectra were recorded between 5–90° 2θ, with a step size of 0.05°/s and a count time of 8 s/step. Diffraction patterns were indexed using Match!3™ software and Powder Data File database (P.D.F. 2000, International Centre of Diffraction Data, Newtown Square, PA, USA).

High-resolution field emission scanning electron microscopy (FE-SEM) was used to determine the surface morphology of Bi_2_O_3_ using a Zeiss Supra-40 microscope (Zeiss, Oberkochen, Germany).

The surface area, pore volume, and pore diameter of the Bi_2_O_3_ were determined by analysis of data points obtained for N_2_ adsorption at −196 °C using a TriStar II analyzer (Micromeritics Instrument Corporation, Norcross, GA, USA) and calculated using the Brunauer–Emmett–Teller (BET) model.

The electrophoretic mobility of Bi_2_O_3_ equilibrated for 24 h in 0.01 M KCl at pH values between 3 and 10 (adjusted with 0.01 or 0.1 M HNO_3_ or KOH) was measured by Laser Doppler Velocimetry coupled with Photon Correlation Spectroscopy (DELSA 440, Beckman Coulter Inc., Brea, CA, USA), and the zeta potential (ζ) was calculated using the Smoluchowski equation [31]. The plot of the ζ potential vs. pH (not shown) provided a PZC for the Bi_2_O_3_ at pH 8.5, in line with the value range reviewed by Ranjan et al. [27] for Bi-based adsorbents.

The surficial chemical composition of the Bi_2_O_3_ was investigated by X-ray photoelectron spectroscopy (XPS) with a PHI 5000 Versaprobe spectrometer (Physical Electronics, Chanhassen, MN, USA) equipped with monochromatic Al Kα X-ray source and operating at 1486.6 eV energy, 15 kV voltage, and 1 mA anode current.

### 2.2. Arsenic Adsorption Experiments

Arsenite and arsenate solutions were prepared by dissolving sodium (meta)arsenite (NaAsO_2_, essay ≥ 90.0, Sigma-Aldrich) and sodium arsenate dibasic heptahydrate (Na_2_HAsO_4_·7H_2_O, essay ≥ 98.0%, Sigma-Aldrich) in doubly deionized water. Preliminary batch tests conducted to study the adsorption of As(III) and As(V) onto Bi_2_O_3_ as a function of pH (adjusted with 0.1 M or 0.01 M NaOH or HNO_3_) indicated a maximum adsorption of As(III) at pH 8 and of As(V) at pH 7 (Figure S1). This can be attributed to the dissociation constants of H_3_AsO_3_ (pK_a1_ = 9.2, pK_a2_ = 12.7, and pK_a3_ = 13.4 [32]) and H_3_AsO_4_ (pK_a1_ = 2.3, pK_a2_ = 6.8, and pK_a3_ = 11.8 [32]), and to the PZC of Bi_2_O_3_ (8.5). These pH values were therefore used in the following adsorption experiments, unless otherwise stated.

All adsorption experiments were carried out in duplicate using 40 mg of Bi_2_O_3_ washed with 10 mL 0.01 M HNO_3_ for 1 h, then with doubly deionized water until the conductivity was below 10 µS m^−1^, indicating the removal of salts from the synthesis. The Bi_2_O_3_ was resuspended in 5 mL of deionized water, sonicated for 15 min (Transsonic T460, Camlab Ltd., Over, UK), then equilibrated with As(III) or As(V) solutions for a final volume of 10 mL on a rotating shaker at 40 rpm and 25 ± 3 °C. The suspensions were subsequently centrifuged for 5 min at 3000 rpm and filtered through a 0.20 μm nylon filter, and the supernatant was analyzed for As(III) or As(V) by the molybdenum blue method proposed by Huang and Fujii [33] unless otherwise stated.

The amount of adsorbed As, *Q_a_* (µmol g^−1^ Bi_2_O_3_) was calculated using the following equation:

$$Q_{a}=\left(C_{0}-C_{e}\right)\times V/m$$

where *C*_0_ (µmol L^−1^) is the initial concentration of As(III) or As(V), *C_e_* (µmol L^−1^) is the equilibrium concentration, *V* (L) is the volume of solution, and *m* (g) is the mass of Bi_2_O_3_.

Adsorption isotherm experiments were conducted at initial As(III) and As(V) concentrations raging between 1.33 and 800 µmol L^−1^ and reaction time of 24 h. The data were fitted to the linear and non-linear Langmuir and Freundlich models, and to the two site Langmuir model, as presented in Table S1 of the Supplementary Material.

Adsorption kinetics experiments were conducted at time intervals ranging between 5 min and 48 h and initial As concentration of 533 µmol L^−1^. Data were fitted to the pseudo-first-order, the pseudo-second-order, and the intra-particle diffusion models, in the forms presented in Table S2.

The competitive effect of coexisting anions on the adsorption of As was studied by equilibrating Bi_2_O_3_ with 533 µmol L^−1^ As(III) or As(V) solution in the presence of 0.01 M or 0.1 M Cl^−^, NO_3_^−^, SO_4_^2−^ or PO_4_^3−,^ and 0.01 M SiO_3_^2−^ for 24 h, and As was determined by hydride generation (HG) coupled with AAS (Perkin-Elmer 4100 equipped with a FIAS 400 hydride generator; Perkin-Elmer Inc., Waltham, MA, USA). The reusability of Bi_2_O_3_ as an adsorbent was evaluated by performing four cycles of As adsorption/desorption. Thus, after equilibration with 533 µmol L^−1^ As(III) or As(V) solution for 24 h, the Bi_2_O_3_ was separated by centrifugation and washed three times with 0.1 M KNO_3_ to remove the arsenic-containing solution. Thereafter, the adsorbent was re-suspended in 10 mL 0.1 M NaOH solution and shaken for 30 min to displace adsorbed As. The supernatants were separated and analyzed for As(III) or As(V), and the Bi_2_O_3_ was dried at 40 °C, resuspended at the initial pH values, and reused.

## 3. Results and Discussion

### 3.1. Characterization of the Bi_2_O_3_

**Figure 1:**
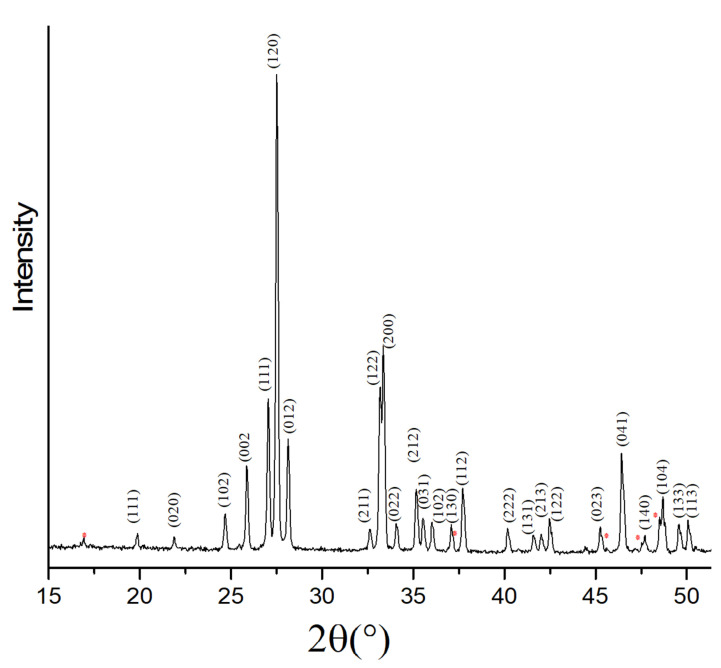
XRD pattern of the synthesized Bi_2_O_3_. Reflection of bismuth oxide is tagged according to Miller indices. Red * represents the reflection not related to bismuth oxide.

Solid-state synthesis is a well-established method to produce size-controlled materials using a mixture of several precursors [34]. In this work, the synthesis of Bi_2_O_3_ was performed with a solid-state approach using Bi(NO_3_)_3_·5H_2_O as the single precursor. The XRD analysis of the obtained Bi_2_O_3_ showed peaks at 2θ = 19.8°, 21.9°, 24.7°, 25.9°, 27.1°, 27.5°, 28.2°, 32.7°, 33.1°, 33.3°, 34.0°, 35.2°, 35.6°, 36.1°, 37.1°, 37.7°, 40.2°, 41.6°, 42.1°, 42.5°, 45.1°, 46.4, 47.1°, 48.6°, 49.5°, and 49.9°. (Figure 1) [35]. This indicated a predominance of the α-phase, according to ICDD database, with a coefficient of similarity of up to 82%, revealing structural and surface defects in the region from 30° 2θ to 50° 2θ, as shown in Figure 1.

**Figure 2:**
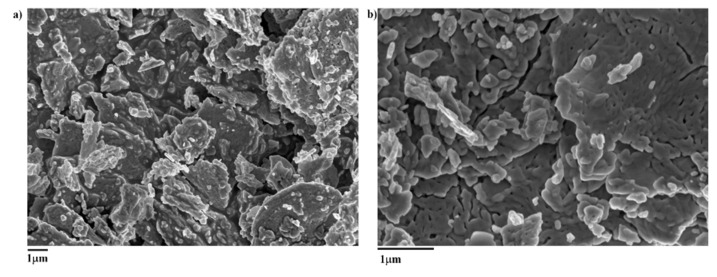
FE-SEM images of freshly synthetized Bi_2_O_3_ at different magnifications ((**a**) 5K, (**b**) 10K).

The FE-SEM analysis showed that the surface of Bi_2_O_3_ was smooth with a diffuse porous structure (Figure 2). Plate-like particles form slit-shaped macro-and mesopores, which may have important implications in the adsorption of As(III) and As(V) from solution.

The Bi_2_O_3_ had a relatively low surface area of 0.83 m^2^ g^−1^ and an average pore diameter of approximately 23.8 nm, which may be attributed to the high temperature employed in the synthesis, previously shown to cause the collapse of micropores, resulting in the formation of mesopores [36]. The shape of N_2_ sorption/desorption isotherms (Figure S2) suggests that the synthesized Bi_2_O_3_ is characterized by a wide distribution of pore sizes due to plate-like particles, in agreement with the FE-SEM observations.

**Figure 3:**
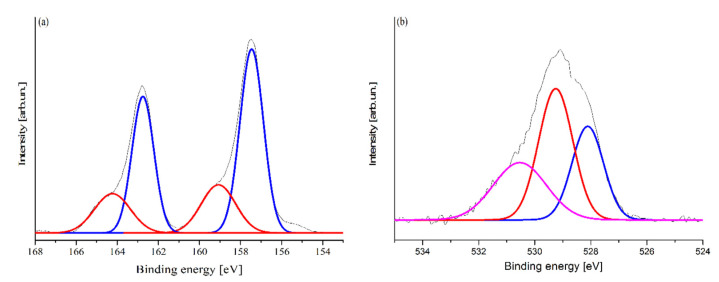
Magnification of high-resolution XPS spectra of synthesized Bi_2_O_3_ in the region (**a**) of Bi 4f_7/2_-Bi 4f_5/2_ (154–168 eV) and (**b**) O 1s (524–534 eV). Purple, red, and blue curves represent the peaks components.

The surface defects of the synthesized Bi_2_O_3_ were further investigated by XPS spectrometry, as reported in Figure 3.

The XPS of bismuth region (Figure 3a) showed the presence of two shouldered peaks, both composed by two different components (164.2 eV, 162.9 eV, 159.1 eV, and 157.4 eV), while oxygen signal (Figure 3b) was composed of three components (530.6 eV, 529.3 eV, and 528.2 eV). According to Shaik et al. [37], bismuth oxides are characterized by two monomodal signal peaks at around 157.9 eV and 163.2 eV. The synthetized Bi_2_O_3_ showed an additional component, probably due to the higher oxidation state of the bismuth species. These species could be induced by the degradation of nitrate groups leading to overoxidation of Bi(III) to high unstable Bi(V) [38,39].

According to this hypothesis, oxygen signal showed three components while pure bismuth oxide showed only at 530 eV with one shoulder at around 529 eV due to hydroxylic functionalities [40]. The additional oxygen peak could be associated with Bi(V) sites included in Bi_2_O_3_ crystalline structure. Furthermore, no appreciable signals in the region of nitrogen (390–410 eV) were detected (Figure S3), supporting the presence of Bi(V) instead of bismuth sub-nitrates species.

### 3.2. Arsenic Adsorption

#### 3.2.1. Adsorption Isotherm Studies

**Figure 4:**
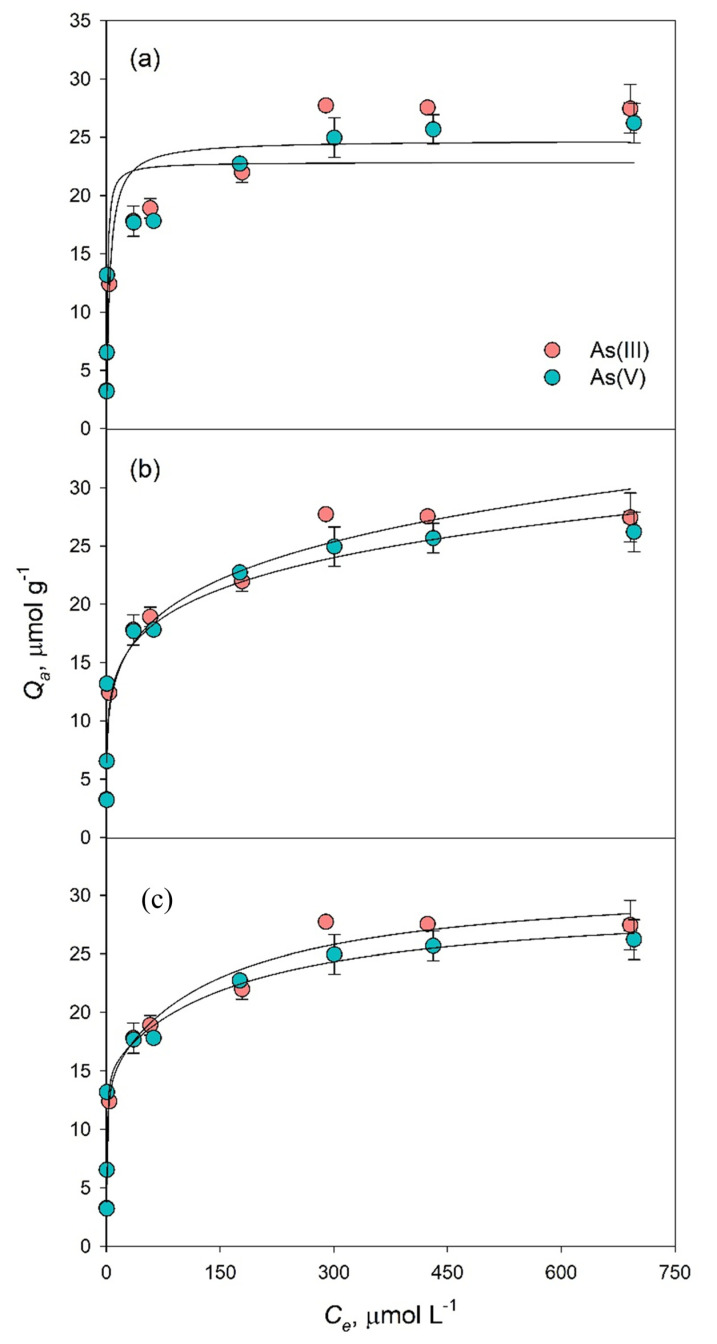
Non-linear Langmuir (**a**), Freundlich (**b**) and two-site Langmuir (**c**) isotherms for As(III) and As(V) adsorption onto Bi_2_O_3_ at pH 8 and 7, respectively, and 25 ± 3 °C. Error bars represent the standard error. Where the error bars are not visible, they are smaller than the size of the symbol.

The adsorption of As was strong at low initial concentrations, with approximately 98% of As(III) and As(V) being adsorbed at *C*_0_ between 13.3 and 53.4 µmol L^−1^ (Figure 4). The adsorption efficiency decreased at higher *C*_0_ to around 14%, representing 27.5 µmol As(III) and 26.2 µmol As(V) g^−1^ Bi_2_O_3_. Despite their generally similar adsorption, As(III) saturated the surface of Bi_2_O_3_ at a lower *C*_0_ than As(V) (400 vs. 800 µmol L^−1^).

We plotted the experimental data of As(III) and As(V) adsorption on Bi_2_O_3_ in non-linearized Langmuir, Freundlich, and two-site Langmuir isotherms (Figure 4), which may help understand the mechanisms controlling the partition of the adsorbate between the liquid and the solid phases at equilibrium. The linearized form of the Langmuir and Freundlich equations are presented in Figure S4.

The Langmuir model is valid for monolayer adsorption on a surface with finite number of identical sites [41], while Freundlich model can be applied to non-ideal adsorption on heterogeneous surfaces and to multilayer sorption [42]. The two-site Langmuir model describes the adsorption of a species on two or more distinct types of sites, which can be described with their own Langmuir expression [43].

The non-linear Langmuir model did not yield a good fitting of the data, as suggested by the difference between the calculated and the experimental values for As(III) and As(V) adsorption (Figure 4a) and by the low R^2^ (Table 1). In most adsorption studies, Langmuir (as well as Freundlich) model is applied in linearized form, since this allows simpler calculation of the equation constants, often yielding apparently good descriptions of the adsorption data. Indeed, the linearized form (Figure S4a) had a high R^2^ also in our case (0.996 and 0.998 for As(III) and As(V), respectively), and a Q_max_ close to experimental values (Table 1), suggesting that As molecules adsorb on the Bi_2_O_3_ surface forming a monolayer. However, linearization of the Langmuir equation is known to bias the regression analysis toward fitting the low *C_e_* values better than the high *C_e_* values [44]. The regression analysis may therefore be strongly affected by small errors in the low aqueous concentration range because of the reciprocal form of the equation. Moreover, the adsorbing sites of the defect-rich material are likely heterogeneous, binding As with different strengths and possibly through more than a single mechanism, hence no mechanistic inferences can be drawn solely from the good fitting of the linearized Langmuir equation. 

The non-linear Freundlich model was a better fit for the experimental data compared to the non-linear Langmuir (Figure 4a,b), giving R^2^ of 0.957 and 0.904 for As(III) and As(V), respectively (Table 1), suggesting that their adsorption mechanism on Bi_2_O_3_ may be controlled by multilayer adsorption on heterogeneous sites. The value of 1/n gives an indication on the affinity between adsorbent and adsorbate, and n between 1 and 10 suggests favourable adsorption. In our case, the high n values for As(III) and As(V) (Table 1) indicated that their adsorption on Bi_2_O_3_ is favourable and helped describe the fast increase in *Q_a_* with increasing *C_e_* at low *C*_0_ values, which gave rise to a highly curved isotherm (Figure 4b). Despite the similar adsorption of As(III) and As(V), the linearized form of the Freundlich model was not suitable to describe As(V) data (Figure S4b), giving R^2^ of 0.767, while the fitting of As(III) adsorption gave an R^2^ of 0.927 (Table 1). On the other hand, the adsorption of As(V) on Bi-impregnated biochar and Bi-impregnated aluminium oxide was well described by the linear Langmuir and linear Freundlich models, respectively [28,30], indicating that the preparation of the adsorbent plays an important role in the adsorption mechanisms.

The two-site Langmuir model assumes that the adsorbent has two types of adsorption sites with different adsorption energies and complies with the three assumptions of the Langmuir model: (i) the adsorption of the molecules forms a monolayer; (ii) the adsorption energy is uniform and remains unchanged; and (iii) there is no interaction between adsorbate molecules [43,45]. Our data fit the two-line Langmuir model (Figure 4c) better than the non-linearized forms of one-site Langmuir and Freundlich equations (Figure 4a,b), giving R^2^ values of 0.987 and 0.920 for the adsorption of As(III) and As(V), respectively. The sum between *Q_max_*_1_ and *Q_max_*_2_ was higher than the *Q_max_* calculated with the non-linear one-site Langmuir equation (Table 1) and was closer to experimental values, suggesting that the two-site Langmuir may be more appropriate for the description of As adsorption on Bi_2_O_3_. While the values of K_L1_ are comparable for the two As forms, the higher *K_L_*_2_ for As(V) compared to As(III) suggests that type II binding sites are more favourable for the adsorption of As(V) than of As(III).

When the adsorption capacity of Bi_2_O_3_ is calculated in terms of µmol m^−2^ from the sum of the two adsorbed layers predicted by the two-sites Langmuir model, the result is of about 36 µmol m^−2^ for As(III) and 34 µmol m^−2^ for As(V), which is in the order of magnitude found by Wang et al. [29] for δ-Bi_2_O_3_, and on average much higher than other metal-oxide based adsorbents (typically 2 to 5 µmol m^−2^ for Fe-(hydr)oxides at the most favourable pH) [46]. The adsorbed concentrations of As(III) and As(V) were much higher than 1.11 µmol g^−1^ obtained for As(V) adsorption onto a hydrous bismuth oxide [47], although smaller than the concentrations adsorbed onto a δ-Bi_2_O_3_ with SSA of 8.99 m^2^ g^−1^ (170.8 and 49.3 µmol m^−2^, respectively) [29]. This very high concentration of adsorbed As was nearly irreversibly retained within the solid [29], hampering its regeneration and reusability, differently from the adsorbent tested in this study (§ 3.2.4). For obtaining adsorbents with larger specific surface area, Bi-doped materials, such as biochar [30] or Al oxides [28], were prepared, reaching SSA values of 190.4 and 130.65 m^2^ g^1^ respectively, with 1.1 and 2.7 µmol m^−2^ of As(III) adsorbed. The adsorption capacity of these Bi-impregnated substrates seemed to be controlled by the amount of Bi incorporated rather than by the specific surface [30].

The high adsorption density for surface unit displayed by bismuth oxides in this study and in [29] increases the interest in developing Bi-based adsorbents for water treatment. It could suggest that specific single layer adsorption might not be the sole adsorption mechanism, in agreement with the better fitting of the adsorption data by Freundlich than Langmuir model in the non linearized form (Figure 4), and by the better performance of two-site than one-site Langmuir equation (Table 1).

#### 3.2.2. Adsorption Kinetic Studies

**Figure 5:**
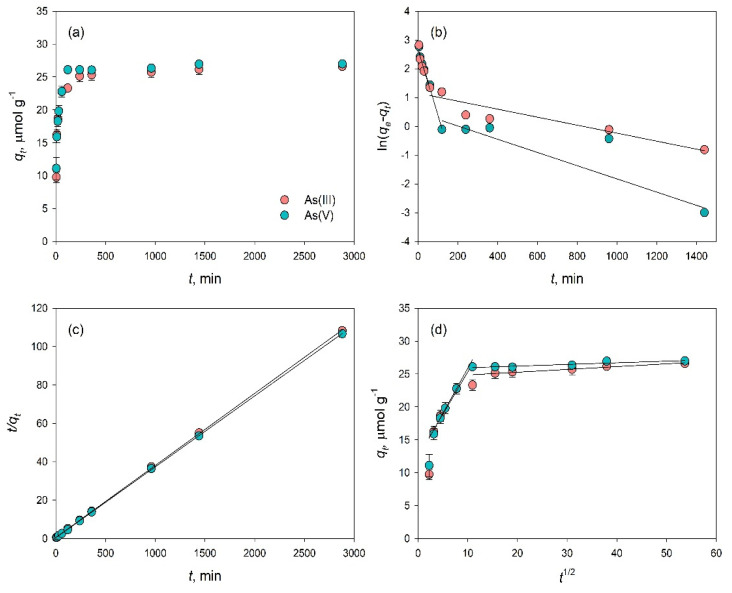
Kinetics of As(III) and As(V) adsorption on Bi_2_O_3_ (4 g/L) at pH 8 and 7, respectively, and 25 ± 3 °C: variation of *q* with time (**a**), pseudo-first-order model (**b**), pseudo-second-order model (**c**), and intraparticle diffusion model (**d**). Error bars represent the standard error. Where the error bars are not visible, they are smaller than the size of the symbol.

Adsorption kinetic experiments showed that As adsorption on Bi_2_O_3_ proceeded rapidly at first, reaching 94% of the total As(III) and As(V) concentration in 4 and 2 h, respectively, then increased slowly until 48 h (Figure 5a). The pseudo-first order model was adequate to describe the adsorption of As(III) for the first 1 h and of As(V) for the first 2 h of adsorption, but beyond that, the data deviated from linearity (Figure 5b). This model gave low R^2^ of 0.732 and 0.770 for As(III) and As(V), respectively, and we therefore divided the two curves, and the calculated parameters are presented in Table 2. However, the inconsistencies between experimental and calculated values of *q_e_* indicated that this model is not the most suitable to describe the adsorption of As on Bi_2_O_3_. The pseudo-second-order kinetics model on the other hand was able to describe the adsorption more precisely, yielding a linear plot for the entire range of adsorption time (Figure 5c), high R^2^ values, and *q_e_* similar to the experimental values, providing the best description of As(III) and As(V) adsorption kinetics among the tested models (Table 2). This suggests that the rate-limiting step for the adsorption of As(III) and As(V) may be controlled by the same mechanism and only slightly changes with time.

In order to verify the importance of intraparticle diffusion as the possible limiting step of As adsorption on Bi_2_O_3_, the data were fitted to the Weber-Morris intraparticle diffusion model [48]. This model assumes that if the plot shows a straight line passing through origin, then the adsorption process is governed by intraparticle diffusion. Our data generated plots exhibiting multiple straight lines that did not pass through the origin (Figure 5d), indicating that intraparticle diffusion is not the major rate limiting step in As multistage adsorption. The curve was thus divided into two distinct linear regions for As(III) and As(V) adsorption, which suggests that the adsorption of As on Bi_2_O_3_ might not be completed in one step, because it is a rather complex process in which more than one mechanism dictates the sorption mechanisms. Similar observations were also made by Zhu et al. for the adsorption of As on Bi-impregnated Al oxide [28] and Bi-impregnated biochar [30], and by Srivastav and co-workers for the adsorption of fluoride [49] and nitrate [50] on hydrous bismuth oxide. The slightly lower rate constants calculated for As(V) than for As(III) (Table 2) support the differences between the adsorption of the two species and the higher affinity of Bi_2_O_3_ for As(III) compared to As(V).

#### 3.2.3. Effect of Competing Anions

**Figure 6:**
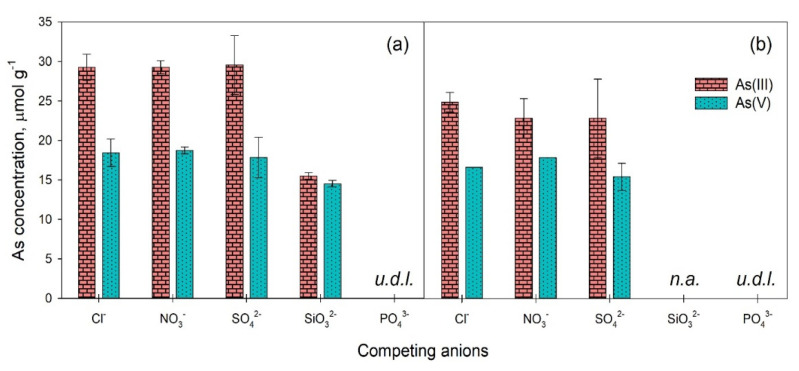
Concentrations of As(III) and As(V) adsorbed on Bi_2_O_3_ (4 g L^−1^) at pH 8 and 7, respectively, and 25 ± 3 °C after 24 h of equilibration in the presence of 0.01 M (**a**) and 0.1 M (**b**) Cl^−^, NO^3−^, SO_4_^2−^, SiO_3_^2−^, and PO_4_^3−^. Error bars represent standard error.

Competing ions had different effects on the adsorption of As(III) and As(V) on Bi_2_O_3_, depending on the species added and their ionic strength, as illustrated in Figure 6. The results showed that the adsorption of As(III) was not hindered by the coexistence of 0.01 M Cl^−^, NO_3_^−^ and SO_4_^2−^. However, 0.01 M SiO_3_^2−^ did reduce the amount of As(III) adsorbed on Bi_2_O_3_ from 27.5 to 15.5 µmol g^−1^ (Figure 4 and Figure 6a). This may be attributed to the similar chemistry between silicic acid (H_4_SiO_4_) and arsenous acid (H_3_AsO_3_), which have similar dissociation constants (pK_a1_ = 9.9 and 9.2, respectively [32]), enabling SiO_3_^2−^ to compete for the same binding sites on the surface of Bi_2_O_3_ as As(III). Increasing the molarity of the competing anions form 0.01 M to 0.1 M resulted in the decrease in the concentration of adsorbed As(III) by 15% in the case of Cl^−^, and by 22% in the case of NO_3_^−^ and SO_4_^2−^ (Figure 6b), suggesting that As(III) adsorption on Bi_2_O_3_ is partially hindered at high background ionic strength.

The adsorption of As(V) was affected by the competing anions to a higher extent compared to As(III), even in the presence of 0.01 M Cl^−^, NO_3_^−^, and SO_4_^2−^, when adsorbed As(V) was reduced from 26.2 (*Q_max_*) to approximately 18.4 µmol g^−1^, while 0.01 M SiO_3_^2−^ decreased retained As(V) to 14.5 µmol g^−1^ (Figure 6a). These results may be attributed to the greater sensitivity of the negatively charged arsenate anions than the nearly undissociated arsenous acid to the formation of an electrostatic barrier by the adsorption of the competing anions. Indeed, the mass transfer of NO_3_^−^ on hydrous bismuth oxide, for example, was shown to occur in the first 40 min of adsorption [51], which is faster than the 2 h estimated for the mass transfer of As(V) on Bi_2_O_3_ (Figure 5d), suggesting that the adsorption of the negatively charged As(V) on Bi_2_O_3_ would be hindered in the presence of some anions because of electrostatic repulsion. However, raising the concentration of Cl^−^, NO_3_^−,^ and SO_4_^2−^ from 0.01 M to 0.1 M affected the amounts of adsorbed As(V) by only 10%, 5% and 14%, respectively (Figure 6b), while the amounts of adsorbed As(III) diminished more markedly, suggesting that As(V) adsorption was less affected by the increase in the background ionic strength compared to As(III). In a previous study conducted with δ-Bi_2_O_3_, Wang et al. [29] also observed a greater interference of competing anions on As(V) than on As(III) adsorption with concentrations of competitors as low as 0.1 and 1.0 mM. Thus, although the adsorption of As(V) was influenced even by low concentrations of Cl^−^, NO_3_^−^, and SO_4_^2^, the decrease was not linear with the increase in the concentration of the competing anions since As(V) bonding took place even in more concentrated saline solutions. On the other hand, As(III) adsorption decreased at the higher electrolyte concentration, but the final adsorbed amount remained greater than that of As(V) in all cases (Figure 6), indicating a better performance of Bi_2_O_3_ in binding the more toxic and generally more difficult to remove As(III), even in a saline environment. The reduction in As adsorption onto Bi_2_O_3_ with high saline concentration may be attributed to the interaction of the different dissolved inorganic species (Cl^−^, NO_3_^−^, SO_4_^2−^, SiO_3_^2−,^ and PO_4_^3−^) with the surfaces of the material [27], consequently competing for the active adsorption sites and deteriorating the capacity of Bi_2_O_3_ to remove As.

The adsorption of As(III) and As(V) in the presence of 0.01 M and 0.1 M PO_4_^3−^ was completely hampered (Figure 6), suggesting that Bi_2_O_3_ had a greater affinity for PO_4_^3−^ than for both As species. Even lower phosphate concentrations significantly decreased As adsorption on δ-Bi_2_O_3_ [29], affecting As(V) adsorption to a larger extent compared to As(III). However, the effect of PO_4_^3−^ on Bi-based compounds employed for the adsorption of As is not routinely determined [28,30,47], which may result in biased indications regarding the efficacy of this material for As removal from complex aqueous media.

#### 3.2.4. Regenerability and Reusability of Bi_2_O_3_

**Figure 7:**
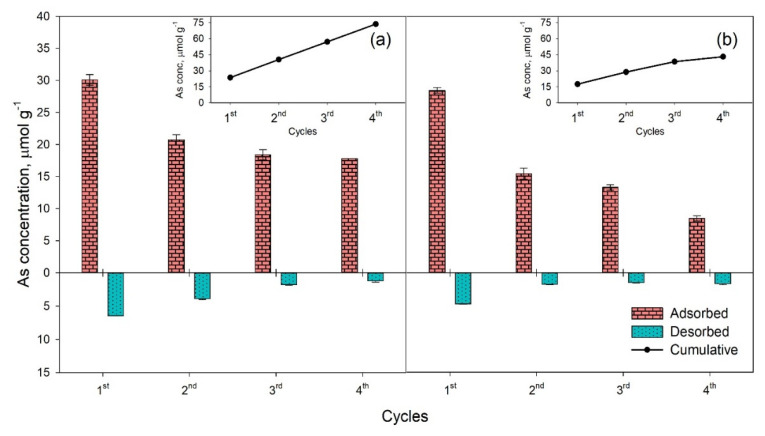
Concentrations of As(III) (**a**) and As(V) (**b**) adsorbed to and desorbed from the surface of Bi_2_O_3_ (4 g L^−1^) at pH 8 and 7, respectively and 25 ± 3 °C in 4 cycles. The inlet graphs show the cumulative amounts of As bound to the adsorbent. Error bars represent standard error.

The possibility to regenerate and reuse the adsorbent greatly enhances its usefulness in water treatment processes. This implies a regeneration step in which all or some of the toxic substance adsorbed is separated from the material, which can be subsequently reused. We studied the removal of As from solution by four adsorption/desorption sequences and observed that the efficiency of Bi_2_O_3_, although decreasing to some extent with each regeneration cycle (Figure 7), remained satisfactory, particularly for As(III), up to the last tested step. The concentrations of adsorbed As(III) and As(V) in the second cycle were 71% and 54% of the amounts adsorbed in the first cycle, respectively. In the third and fourth cycle, the concentrations of As(III) adsorbed remained relatively constant, while As(V) adsorbed after the last regeneration cycle was approximately 30% of the initial amount. The reduction in As adsorption may be due to its gradual stabilization on the surface of Bi_2_O_3_, as indicated by the incomplete removal with NaOH (Figure 7), therefore rendering part of the active sites unavailable for subsequent As binding. Indeed, washing with 0.1 M KNO_3_ and NaOH resulted in the removal of 6.5 µmol As(III) g^−1^ and 11.0 µmol As(V) g^−1^ in the first cycle (Figure 7), representing 21% and 39% of the adsorbed amounts, respectively, which may be constituted mainly by the most weakly-bound portion of As anions. The concentrations of As retained in each cycle, however, always exceeded those previously desorbed, which resulted in a linear accumulation of As(III) (R^2^ = 1.000) and As(V) (R^2^ = 0.969) on the surface of the material (Figure 7), indicating that the incomplete As removal was compensated by new adsorbing sites becoming available after the regeneration steps. Indeed, the decreasing of the As adsorbed at each cycle was paralleled by a reduction in the capacity of NaOH to displace bound As, as desorbed As(III) concentrations decreased from 6.5 to 1.2 µmol g^−1^ (Figure 7a), and As(V) decreased from 11.0 to 3.9 µmol g^−1^ (Figure 7b). Our results are in line with those obtained by Zhu et al. [28] for the amounts of As removed from Bi-impregnated Al_2_O_2_ with 0.1 M HCl, which decreased with each regeneration cycle. Although a smaller fraction of the adsorbed As was removed at each regeneration step, the material continued to adsorb substantial amounts of both As specie. This resulted in a progressive build-up of As on the adsorbing substrate, which remained linear for As(III) after four regeneration cycles and started bending after the third one for As(V) (Figure 7).

The efficiency of Bi_2_O_3_ to adsorb and retain As(III) was higher compared to As(V) throughout the four regeneration cycles, as suggested by the total concentrations of the two species (73.7 µmol g^−1^ vs. 43.1 µmol g^−1^, Figure 7). This further confirms the higher affinity of Bi_2_O_3_ for As(III) compared to As(V), observed in the adsorption studies and examining the competition with other ions (Figure 4 and Figure 6). The building up of bound As, however, exceeding the *Q_max_* observed in the adsorption studies (Figure 4), suggests that the washing of the substrate with 0.1 M KNO_3_ and desorption with 0.1 M NaOH may interact with the defective surface of the Bi_2_O_3_, consequently activating new sites available for As adsorption, or that other mechanisms different from adsorption, such as surface precipitation, might contribute to As retention.

### 3.3. In-Solution Behaviour of Defective Bi_2_O_3_ and Implication for its Usage in Water Treatment

Bi_2_O_3_ could be easy tailored through surface modifications both during and after its synthesis [26,52,53]. This property is very attractive for electrochemical applications but implies unneglectable consequences during contaminant adsorption in real groundwater and wastewater environments. When the yellow-colored defective Bi_2_O_3_ is interacted with nitric acid, the solid color turned from light yellow to white. This was reasonably due to the surface modification induced by the formation of bismuth subnitrate sites.

This phenomenon leads to the uncontrolled variation of Bi_2_O_3_ adsorption properties, its extent depending on the acid concentration, and time of interaction, thus making the surface properties, and hence its adsorption properties, dependent on the chemistry of the solution to be treated. Similarly, defects could be also induced by the washing with concentrated alkali, promoting the formation of hydroxyl functions on the surface of the material. An original defectiveness structure of oxygen sites in Bi_2_O_3_ reticule further exploited the surface tailoring as reported by Gökağaç et al. [54].The surface modification of Bi_2_O_3_ is reasonably accounted for by the adsorption performances after several cycles.

For both As(III) and As(V) species, the adsorption process took place very fast in the first 4 and 2 h, respectively, reaching a stable constant value after 16h. The initial fast adsorption of arsenic species might be attributed to the fine structures of Bi_2_O_3_ combined with the surface modifications related to the disappearance of Bi(V) sites, supporting the hypothesis that they are related to a defective structure and not to a well defined phase.

**Figure 8:**
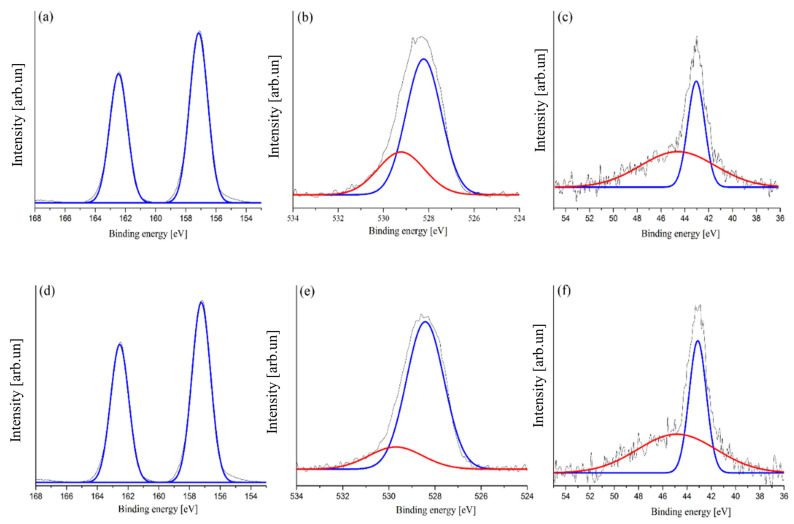
Magnification of high-resolution XPS spectra of Bi_2_O_3_ after the adsorption of As(V) in the region (**a**) of Bi 4f_7/2_-Bi 4f_5/2_ (154–168 eV), (**b**) O 1s (524–534 eV), and (**c**) As 3d_5/2_-3d_3/2_ (54–36 eV) and after the adsorption of the As(III) in the region (**d**) of Bi 4f_7/2_-Bi 4f_5/2_ (154–168 eV), (**e**) O 1s (524–534 eV), and (**f**) As 3d_5/2_-As 3d_3/2_ 1s (54–36 eV). Red and blue curves represent the peaks components.

The XPS spectra acquired after the fourth adsorption cycle (Figure 8) showed an appreciable simplification of bismuth and oxygen related signals. This could be one explanation for the general decrement in the adsorption of As(III) and As(V) with each regeneration cycle (Figure 7). However, the accumulation of As adsorbed may be attributed to the exposure of new binding sites with each cycle of desorption with NaOH and washing with KNO_3_.

After both As(V) and As (III) adsorption, Bi 4f_7/2_ and Bi 4f_5/2_ were composed by only one component centered at 157.3 eV and 164.1 eV, respectively, while oxygen signal showed two components at 529. 3 eV and 528.1 eV. Curiously, the signals of adsorbed As(V) and As(III) were both composed of two components centered at 44.7 eV and 43.0 eV, corresponding to As(V) and As(III), respectively [53]. The redox of arsenic compounds during adsorption process is generally due to electrochemical active sites as in the case of Fe(II) or Mn related compounds [55,56], but in the case of Bi_2_O_3_ could be imputable to oxidative power of Bi(V) sites embedded into Bi_2_O_3_ [57] leading to oxidation of As(V).

This mechanism underwent a progressive deactivation caused by the high instability of Bi(V) sites together with the irreversibility of its reduction, leading to the progressive decrement of adsorption performances.

The combination of surface instability and defects insertion could represent a boost for the application of B_2_O_3_ as tool for treating high metal contaminated water waste-streams even if it is required a fine tuning for improving its duration.

## 4. Conclusions

Bi_2_O_3_ was synthesized by a solid-state reaction method that induced the formation of Bi(V) surface modifications in the defective structure of a highly reactive material. This new material was successfully tested for the removal of arsenite and arsenate from aqueous solutions. Both As species adsorbed rapidly, reaching equilibrium within 2 h for As(III) and 4 h for As(V), and the whole kinetics were well described by the pseudo-second order model, indicating that the rate-limiting step for the adsorption of both As species may be controlled by the same mechanism. The defective Bi_2_O_3_ adsorbed up to 33.1 and 31.6 µmol m^−2^ As(III) and As(V), respectively, and the adsorption isotherms were best described by the two-sites Langmuir model. We showed that As adsorption onto Bi_2_O_3_ is not hampered in the presence of chloride, nitrate, and sulfate but is moderately decreased by the presence of silicate and at high ionic strength. However, phosphate efficiently competed with both As species, impeding their removal from solution, which must be taken into consideration when using Bi-based substrates for the treatment of As-contaminated water or wastewater. This adsorbent can be regenerated and reutilized several times. It was still able to remove As from the solution after four cycles of regeneration, even if partial deactivation occurred. The aforementioned characteristics proved the suitability of Bi_2_O_3_ as a reliable and effective adsorbent for As removal from water. The possibility to adsorb both As(III) and As(V) in comparable amounts, with similar kinetics and with no redox pre-treatments, on a substrate that can be regenerated and reused makes Bi_2_O_3_ a promising and innovative material for the treatment of As-contaminated water. 

## Figures and Tables

**Table 1:** Isotherm parameters obtained for non-linear and linear Langmuir and Freundlich models and for the two-site Langmuir model for the adsorption of As(III) and As(V) onto Bi_2_O_3_.

Species	Langmuir Parameters					
	Non-Linear Model			Linear Model		
	*Q_max_* (µmol g^−1^)	*K_L_* (L mol^−1^)	R^2^	*Q_max_* (µmol g^−1^)	*K_L_* (L mol^−1^)	R^2^
As(III)	24.739	0.242	0.873	27.983	0.070	0.996
As(V)	22.863	0.843	0.812	26.439	0.075	0.998
	Freundlich parameters					
	Non-linear model			Linear model		
	*K_F_*	*n*	R^2^	*K_F_*	*n*	R^2^
As(III)	8.264	5.086	0.957	6.554	4.068	0.927
As(V)	8.926	5.767	0.904	7.418	4.771	0.767
	Two-site Langmuir parameters					
	Type I site			Type II site		
	*Q_max_*_1_ (µmol g^−1^)	*K_L_*_1_ (L mol^−1^)		*Q_max_*_2_ (µmol g^−1^)	*K_L_*_2_ (L mol^−1^)	R^2^
As(III)	16.858	0.007		14.588	1.233	0.987
As(V)	14.463	0.006		15.213	1.738	0.920

*Q_max_*, *Q_max_*_1_, and *Q_max_*_2_ (µmol g^−1^)—maximum amount of analyte that can bind to the Bi_2_O_3_ as a monolayer or on type 1 and type 2 sites, respectively; *K_L_*, *K_L_*_1_, and *K_L_*_2_ (L mol^−1^)—Langmuir affinity constants of the monolayer and the type 1 and type 2 sites. *K_F_*—Freundlich constant, representing adsorption capacity at unitarian concentration at equilibrium. *n*—empirical constant, indicating the adsorption intensity of the system. R^2^—coefficient of determination.

**Table 2:** Kinetic parameters for the adsorption of As(III) and As(V) on Bi_2_O_3_.

Species	Pseudo-First-Order					
	*k*_1_ (min^−1^)	*q_e_*_1_ (µmol g^−1^)	R^2^	*k*_2_ (min^−1^)	*q_e_*_2_ (µmol g^−1^)	R^2^
As(III)	0.055	496	0.910	0.004	14.53	0.896
As(V)	0.054	536	0.989	0.005	3.13	0.763
	Pseudo-second-order					
	*k* (g/µmol/min)		*q_e_* (µmol g^−1^)	R^2^		
As(III)	0.0027		26.647	0.999		
As(V)	0.0031		27.076	1.000		
	Intraparticle diffusion					
	*k_i_*_1_ (µmol/g/min^0.5^)		R^2^	*k_i_*_2_ (µmol/g/min^0.5^)		R^2^
As(III)	1.367		0.969	0.0425		0.869
As(V)	1.281		0.981	0.0247		0.871

*k*_1_, *k*_2_ (min^−1^), *k* (g/µmol/min), *k_i_*_1_, *k_i_*_2_ (µmol/g/min^0.5^)—adsorption rate constants. *q_e_*_1_, *q_e_*_2_, *q_e_* (µmol g^−1^)—concentration of As(III) or As(V) adsorbed on Bi_2_O_3_ at equilibrium. t (min)—time. R^2^—coefficient of determination.

## Data Availability

The data presented in this study are available on request from the corresponding author. The data are not publicly available due to institutional and national data sharing restrictions.

## Supplementary Materials

The following are available online at https://www.mdpi.com/article/10.3390/toxics9070158/s1, Figure S1. Effect of initial pH on the adsorption of As(III) and As(V) onto Bi_2_O_3_. Experimental conditions: initial As(III) and As(V) concentration—40 mg As L^−1^; adsorbent dose—4 g L^−1^; temperature—25 ± 3 °C; time—24 h, Figure S2. N_2_ adsorption-desorption isotherms of synthesized Bi_2_O_3_, Figure S3. XPS spectra of Bi_2_O_3_ after washing with HNO_3_ 0.1 M, Figure S4. Linear Langmuir (a) and Freundlich (b) isotherms for As(III) and As(V) adsorption onto Bi_2_O_3_ (4 g/L) at pH 8 and 7 respectively, and 25 ± 3 °C, Table S1. Linear and non-linear forms of the Langmuir and Freundlich models, and to the two site Langmuir model used to fit the adsorption of As(III) or As(V) on Bi_2_O_3_, Table S2. Pseudo-first-order, pseudo-second-order, and intraparticle diffusion models used to fit the kinetic data of As(III) and As(V) adsorption on Bi_2_O_3_.
