# Supplementary material for: Defective Bismuth Oxide as Effective Adsorbent for Arsenic Removal from Water and Wastewater
Source: Toxics. 2021 Jul 2;9(7):158. doi: 10.3390/toxics9070158 (PMC8309858; doi:10.3390/toxics9070158)
Supplement: Supplementary file 1 [file toxics-09-00158-s001.zip › toxics-1275202-supplementary.pdf]

toxics

Article

Supplementary Materials: Defective Bismuth Oxide as Effective Adsorbent for Arsenic Removal from Water and Wastewater

Ramona Balint, Mattia Bartoli, Pravin Jagdale, Alberto Tagliaferro, Abdul Samad Memon, Massimo Rovere and Maria Martin

1. Characterization of Bi_2O_3

1.1. BET

The N_2 adsorption increased slowly at low relative pressures and then raised rapidly near p/p0 = 0.8 (Figure 1S). According to IUPAC and Lowell et al. [1], this is defined as a type II isotherm with the hysteresis loop classified as an H3 loop, which is characteristic for monolayer coverage followed by multilayering at high relative pressures. This suggests that the synthesized Bi_2O_3 is characterized by a wide distribution of pore sizes due to plate-like particles, which form slit-shaped macro- and mesopores [2]. This was supported by the low surface area of 0.83 $m^2 g^{-1}$ and the average pore diameter of approximately 23.8 nm, which may be attributed to the high temperature employed in the synthesis of Bi_2O_3, previously shown to cause the collapse of micropores, resulting in the formation of mesopores [3].

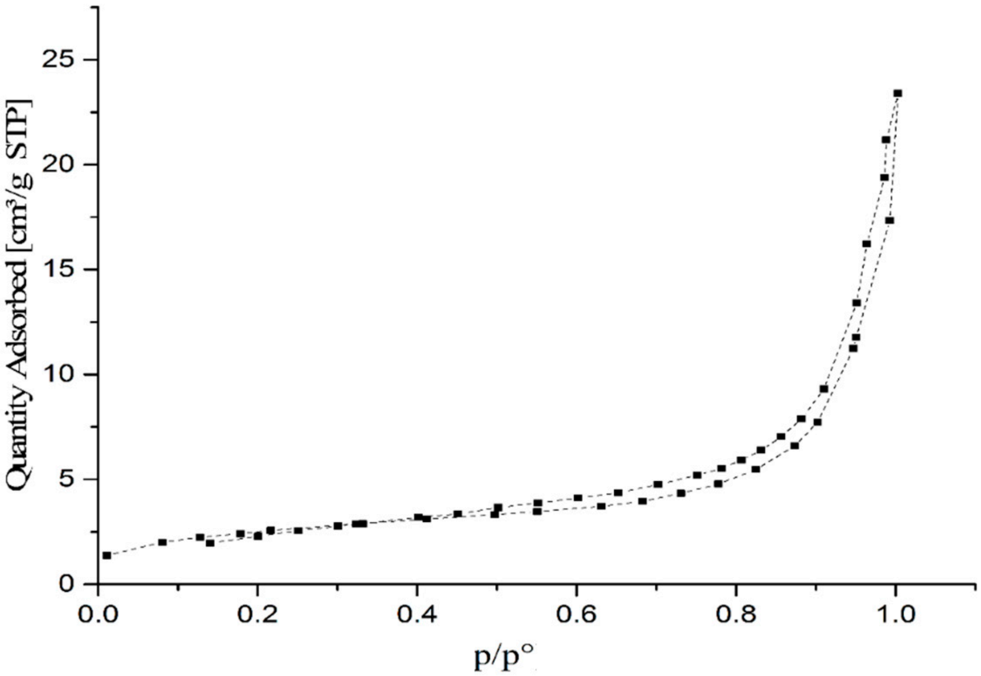

Figure S1. N_2 adsorption-desorption isotherms of synthesized Bi_2O_3.

1.2. XPS

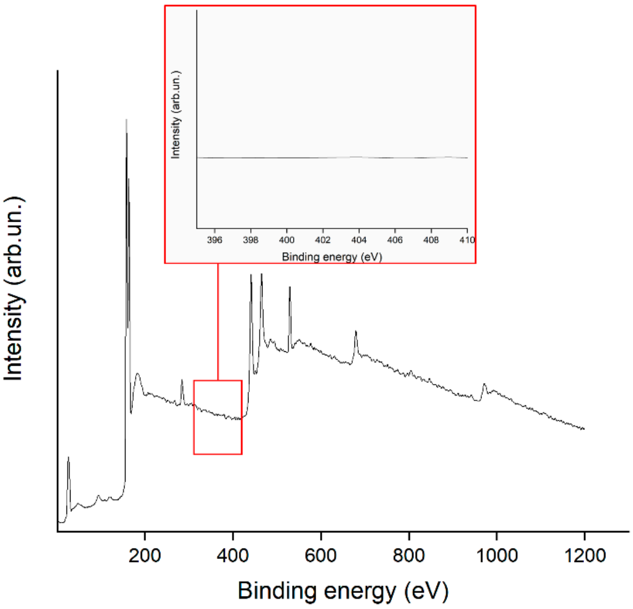

Figure S2. XPS spectra of Bi_2O_3 after washing with HNO_3 0.1 M.

2. Adsorption experiments

2.1. Adsorption isotherms

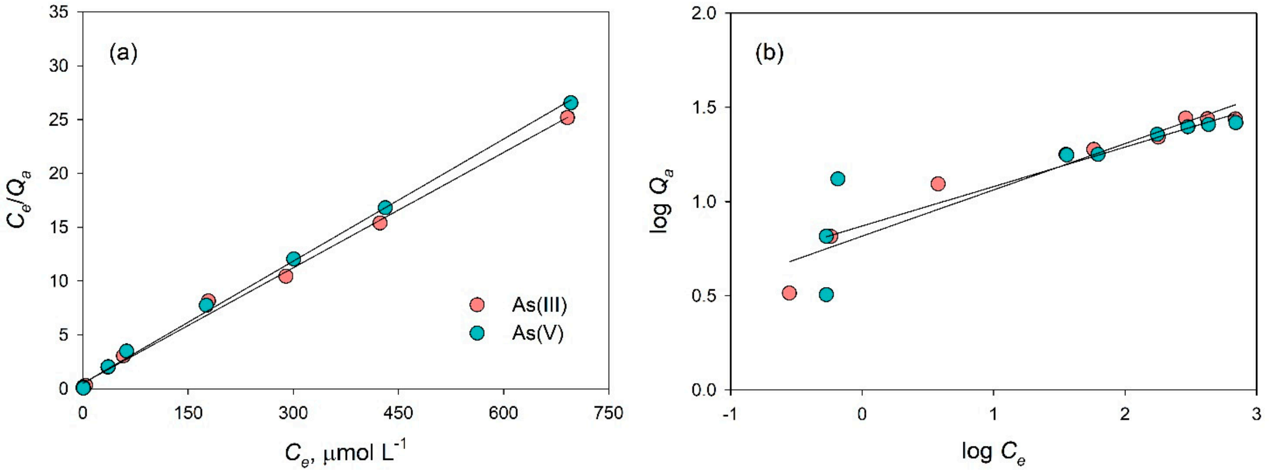

Figure S3. Linear Langmuir (a) and Freundlich (b) isotherms for As(III) and As(V) adsorption onto Bi_2O_3 (4 g/L) at pH 8 and 7 respectively, and 25 ± 3 °C.

Table S1. Linear and non-linear forms of the Langmuir and Freundlich models, and to the two site Langmuir model used to fit the adsorption of As(III) or As(V) on Bi_2O_3.

Isotherm	Non-linear form	Linear form
Langmuir	$Q_a = \frac{Q_{max}\, K_L\, C_e}{1 + K_L\, C_e}$	$\frac{C_e}{Q_a} = \frac{C_e}{Q_{max}} + \frac{1}{K_L Q_{max}}$
Freundlich	$Q_a = K_F C_e^{\frac{1}{n}}$	$\log Q_a = \log K_F + \frac{1}{n} \log C_e$
Two site Langmuir	$Q_a = \frac{Q_{max}\, K_{L1}\, C_e}{1 + K_{L1}\, C_e} + \frac{Q_{max}\, K_{L1}\, C_e}{1 + K_{L1}\, C_e}$	

Q_a (µmol g^{-1}) - concentration of As(III) or As(V) adsorbed on Bi_2O_3. Q_{max}, Q_{max1} and Q_{max2} (µmol g^{-1}) - maximum amount of analyte that can bind to the Bi_2O_3 as a monolayer or on type 1 and type 2 sites respectively. C_e (µmol mL^{-1}) - equilibrium

concentration. K_L, K_{L1} and K_{L2} (L mol^{-1}) - Langmuir affinity constants of the monolayer and the type 1 and type 2 sites. K_F - Freundlich constant, representing adsorption capacity at unitarian concentration at equilibrium. 1/n - empirical constant, indicating the adsorption intensity of the system.

2.2. Adsorption kinetics

Table S2. Pseudo-first-order, pseudo-second-order and intraparticle diffusion models used to fit the kinetic data of As(III) and As(V) adsorption on Bi_2O_3.

Model	Equation
Pseudo-first-order	$q_t = q_e(1 - e^{-k_1 t})$
Pseudo-second-order	$q_t = k_2 {q_e}^2 t/(1 + k_2 q_e t)$
Intraparticle diffusion	$q_t = k_{id} \times t^{0.5} + C$

q_t (μmol g^{-1}) - concentration of As(III) or As(V) adsorbed on Bi_2O_3 at any time t. q_e (μmol g^{-1}) - concentration of As(III) or As(V) adsorbed on Bi_2O_3 at equilibrium. t (min) – time. k_1 (min^{-1}), k_2 (g/μmol/min), k_{id} (μmol/g/$min^{0.5}$) - adsorption rate constants. C – constant corresponding to the thickness of boundary layer.

References

1. S. Lowell, J.E. Shields, M.A. Thomas, M. Thommes, Characterization of porous solids and powders: surface area, pore size and density, Springer Science & Business Media, 2012.
2. Awoke, Y.; Chebude, Y.; Díaz, I. Controlling Particle Morphology and Pore Size in the Synthesis of Ordered Mesoporous Materials. Molecules 2020, 25, 4909. https://doi.org/10.3390/molecules25214909
3. Lan, X., Jiang, X., Song, Y., Jing, X. & Xing, X. (2019). The effect of activation temperature on structure and properties of blue coke-based activated carbon by CO_2 activation. Green Processing and Synthesis, 8(1), 837-845. https://doi.org/10.1515/gps-2019-0054.
